# Evidence that phospholipase C is involved in the antitumour action of NSC768313, a new thieno[2,3-b]pyridine derivative

**DOI:** 10.1186/s12935-016-0293-6

**Published:** 2016-03-10

**Authors:** Jóhannes Reynisson, Jagdish K. Jaiswal, David Barker, Stacey A. N. D’mello, William A. Denny, Bruce C. Baguley, Euphemia Y. Leung

**Affiliations:** Auckland Cancer Society Research Centre, University of Auckland, Private Bag 92019, Auckland, 1142 New Zealand; Molecular Medicine and Pathology Department, University of Auckland, Private Bag 92019, Auckland, 1142 New Zealand; School of Chemical Sciences, University of Auckland, Auckland, New Zealand

**Keywords:** Cancer, Thieno[2, 3-b]pyridine, Phospholipase C, Paclitaxel, Membrane blebbing, Synergy, Cell cycle arrest

## Abstract

**Background:**

The thieno[2,3-*b*]pyridines were discovered by virtual high throughput screening as potential inhibitors of phospholipase C (PLC) isoforms and showed potent growth inhibitory effects in National Cancer Institute’s human tumour cell line panel (NCI60). The mechanism of the anti-proliferative activity of thieno[2,3-*b*]pyridines is explored here.

**Objectives:**

We aimed to investigate the basis for the anti-proliferative activity of these thieno[2,3-*b*]pyridines and to determine whether the cellular inhibition was related to their inhibition of PLC.

**Methods:**

Four breast cancer cell lines were used to assess the anti-proliferative effects (IC_50_ values) of six representative thieno[2,3-*b*]pyridines. The most potent compound (derivative **3**; NSC768313), was further studied in MDA-MB-231 cells. DNA damage was examined by γH2AX expression level, and cell cycle arrest by flow cytometry. Cell morphology was examined by tubulin antibody staining. The growth inhibitory effect of combination treatment with derivative **3** and paclitaxel (tubulin inhibitor), doxorubicin (topoisomerase II inhibitor) or camptothecin (topoisomerase I inhibitor) was evaluated. A preliminary mouse toxicity assay was used to evaluate the pharmacological properties.

**Results:**

Addition of the thieno[2,3-*b*]pyridine derivative **3** to the MDA-MB-231 cells induced G2/M growth inhibition, cell cycle arrest in G2-phase, membrane blebbing and the formation of multinucleated cells. It did not induce DNA damage, mitotic arrest or changes in calcium ion flux. Combination of derivative **3** with paclitaxel showed a high degree of synergy, while combinations with doxorubicin and camptothecin showed only additive effects. A mouse pharmacokinetic study of derivative **3** showed that after intraperitoneal injection of a single does (10 mg/Kg), the C_max_ was 0.087 μmol/L and the half-life was 4.11 h.

**Conclusions:**

The results are consistent with a mechanism in which thieno[2,3-*b*]pyridine derivatives interact with PLC isoforms (possibly PLC-δ), which in turn affect the cellular dynamics of tubulin-β, inducing cell cycle arrest in G2-phase. We conclude that these compounds have novelty because of their PLC target and may have utility in combination with mitotic poisons for cancer treatment.

## Background

The phospholipase C (PLC) family comprises a series of enzymes [1] which regulate many cellular growth functions, making them interesting targets for cancer therapy [2, 3]. The PLC isozymes are membrane bound proteins that hydrolyse phosphatidylinositol 4,5-diphosphate (PIP2) and generate two intracellular products: diacylglycerol (DAG) and inositol 1,4,5-triphosphate (IP3) [4–6]. PLC is critical to cofilin regulation of actin polymerization which triggers the chemotaxis of breast carcinoma cells to epidermal growth factor [7]. There are six subfamilies of the mammalian pi-PLC super-family, which are classified as PLC-β, PLC-γ, PLC-δ, PLC-ε, PLC-ζ and PLC-η. In breast cancer, PLC-δ1 and PLC-δ3 expression levels were positively correlated with disease staging or tumor size [8]. Knockdown of PLC-δ induced cell rounding, surface blebbing and nuclear fragmentation, reduced cell proliferation and migration in breast cancer cell line MDA-MB-231 [8].

**Fig. 1:**
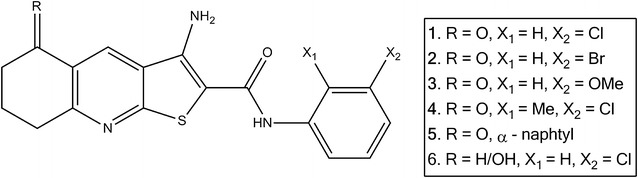
The structures of the thieno[2,3-*b*]pyridine derivatives **1–6**

The thieno[2,3-*b*]pyridines were initially discovered as potential inhibitors of PLC isoforms by virtual high throughput screen (vHTS) against the available crystal structure of the PLC-δ1 isoform [2]. In vitro testing revealed that the most potent analogues had growth inhibitory activity at low nanomolar concentrations against the National Cancer Institute’s NCI60 human tumour cell line panel [9–12]. They were also active against a series of breast cancer cell lines [13]. The structures of the six derivatives are shown in Fig. 1.

Triple-negative breast cancers lack functional oestrogen, progesterone and HER2 receptors, and new effective targeted therapies are required for this subset [14]. The sensitivity of two triple-negative breast cancer cell lines [13] to thieno[2,3-*b*]pyridines prompted our further investigation of this series, firstly to explore the activity of further thieno[2,3-*b*]pyridine analogues, secondly to characterise the cellular basis their anti-proliferative activity, and thirdly to determine whether adequate plasma concentrations of the most active compound are achieved after in vivo administration.

## Methods

### Cell culture

Culture conditions have been described in detail previously [15]; human breast cancer cell lines MCF-7, T47D, MDA-MB-468, MDA-MB-231, human epithelial carcinoma cell line A431 and human colon cancer cell line HCT116 were purchased from the American Type Culture Collection (ATCC). Cells were grown in α-MEM containing 5 % fetal calf serum, insulin/transferrin/selenium supplement, added according to the manufacturer’s instructions (Roche), as well as penicillin/streptomycin (100 U/ml and 100 μg/ml, respectively).

### Cell proliferation assay

As described in detail previously [16], cell proliferation was measured using a thymidine incorporation assay by seeding 3000 cells in each well using 96 well plates with varying concentrations of inhibitors for 3 days. Experiments were performed in triplicate with a minimum of two experimental repeats. Briefly, ^3^H-thymidine (0.04 µCi) was added to each well and incubated for 5 h, after which the cells were gathered onto glass fibre filters using an automated TomTec harvester. Filters were incubated with Betaplate Scint and thymidine incorporation determined with Trilux/Betaplate counter showing the percentage of cells incorporated with ^3^H-thymidine into the DNA helix.

### Calcium flux assay

A431 cells were plated onto 96 wells until confluent. Cells were loaded with 5 µM Fluo-4 acetoxymethyl ester with 0.003 % pluronic acid and 2.5 mM Probenecid (Molecular Probes) for 30 min at 37 °C, then washed with buffer and incubated for another 30 min. Epidermal growth factor (EGF) (400 ng/ml) was used as an activator. EGF alone or with derivative **3** were added to A431 cells to determine the inhibitory activity on calcium flux of derivative **3**, and compared with buffer (negative control) and 1 µg/ml ionomycin (positive control). Fluorescence signal was recorded from the bottom of the plate at 1 s intervals (3 wells per/s) using an EnSpire 2300 Multimode Plate Reader (Perkin-Elmer) at excitation wavelength of 494 nm and emission wavelength of 506 nm (25 °C, 3 mm measurement height, 50 flashes). Baseline fluorescence was recorded for 10 s, after which buffer alone, EGF alone or with drug, was added and fluorescence was captured for a further 45 s. Next, ionomycin was added and fluorescence was recorded for another 45 s. Three independent experiments were performed.

### Microtubule imaging

Cells were grown on a glass slide with and without inhibitors, fixed by 4 % paraformaldehyde, permeabilised by 0.5 % Triton X-PBS and incubated with 5 % BSA/PBS containing FITC-labelled anti-tubulin antibody. Microtubule arrangement was monitored by fluorescence staining of microtubules using anti-tubulin and analysed by fluorescent microscopy using Floid imaging station (460× magnification). Nucleus was stained with 2 μg/ml Hoechst stain (Life Technology) for 10 min at room temperature. Hoechst stain emits blue fluorescence when bound to double strand DNA.

### Flow cytometric analysis

As described in detail previously [17], cells (10^6^ cells per well) were grown in 6 well plates and incubated with inhibitors for the indicated time. Cells were harvested, washed and resuspended in 1 ml of blocking buffer (1 % FCS/PBS), and incubated with antibody to γ-H2AX (phosphorylated Ser139) (Millipore, USA) in blocking buffer (1:500 dilution) at room temperature for 2 h. Cells were washed, incubated with goat anti-mouse Alex 488 Fab fragment secondary antibody (Invitrogen, New Zealand) (1:400 in blocking buffer for 1 h, at room temperature; dark), washed and resuspended in 1 ml of blocking buffer containing RNase (1 μg/ml) and propidium iodide (PI) (10 μg/ml) for 30 min at room temperature. Cells were analysed in a Becton–Dickinson LSRII and profiles were analysed with ModFit LT 3 software.

### Immunoblotting

As described in detail previously [15] cells were grown to log-phase, washed twice with ice-cold PBS, and lysed in SDS lysis buffer according to the manufacturer’s protocol (Cell Signalling Technology, Danvers, MA, USA). Protein concentration was quantified using the BCA protein assay reagent bicinchoninic acid (Sigma, New Zealand). Cell lysates containing 25 μg of protein were separated by SDS-PAGE and transferred to PVDF membranes (Millipore). Membranes were immunoblotted with antibodies against to γ-H2AX (phosphorylated Ser139) (Millipore, USA), and actin (Millipore), using SuperSignalWest Pico (Thermo Scientific, Waltham,MA, USA) chemiluminescence reagents. Antibody reactivity was visualized using the chemiluminescence detection system by Fujifilm Las-3000.

### Data analysis

As described previously for everolimus and other inhibitors [15], we employed the Bliss additivism model [18] to classify the drug combinations as additive, synergistic or antagonistic. A theoretical curve was calculated for combined inhibition using the equation *E*_bliss_ = (*E*A + *E*B) − (*E*A × *E*B), where *E*A and *E*B are the fractional inhibitions obtained by drug A alone and drug B alone at specific concentrations. *E*_bliss_ is the fractional inhibition that would be expected if the effects of the two drugs were additive. The difference (Bliss) in the experimentally measured fractional inhibition (*E*_xpt_) and *E*_bliss_ are defined as synergism (*E*_xpt_ < *E*_bliss_), additive (*E*_xpt_ = *E*_bliss_), and antagonism (*E*_xpt_ > *E*_bliss_). Bliss = 0 would indicate the combination is additive; Bliss > 0 would indicate the percentage increase in maximal inhibition above additivity (synergy); and Bliss < 0 would indicate the percentage decrease in maximal inhibition below additivity (antagonism). The data were analysed using a one-way ANOVA coupled with multiple comparisons versus treatment control applying the Holm-Sidak method correction, where p < 0.05 denotes a statistically significant difference.

### Mouse plasma pharmacokinetics

Male CD-1 mice were obtained from Vernon Jansen Unit (University of Auckland) and housed in cages with a 12-hour light/dark cycle and received sterilized food and water ad libitum. Studies and procedures were conducted with prior approval of the Ethics Committee of the University of Auckland (AEC approval number 1190). Derivative **3** was prepared in 20 % HP-β CD as a fine yellow suspension, and administered (at 10 mg/Kg) as intraperitoneal bolus injection. Blood samples were collected via a terminal cardiac bleeding (n = 3 mice/time) at 0.167, 0.5, 1, 2 and 6 h post dose. Blood was collected in ice cold K_2_-EDTA tubes and kept on ice. All samples were centrifuged at 6500 rpm for 5 min at room temperature, obtained plasma were stored at −80 °C until analysed by LC–MS/MS.

Briefly, plasma samples were assayed for derivative **3** using liquid chromatography-tandem mass spectrometry (LC–MS/MS) method. Briefly, derivative **3** was spiked in the drug mouse plasma to obtain a concentration 0.003, 0.03, 0.3, 3, 10 and 30 µM to construct a calibration curve. Derivative **1** (a structural analogue of **3**) was used as an internal standard in LC–MS/MS assay at a concentration of 0.5 μM added to acetonitrile. Drug was extracted from plasma using direct protein precipitation with ice cold acetonitrile (1:4 v/v) and quantified by peak area ratio of 3 versus 1 using a calibration curve prepared with derivative **3** as outlined above. LC–MS/MS used was Agilent 6410b with multimode ion source operated in atmospheric pressure chemical ionization (APCI) positive mode attached to an Agilent 1200 series HPLC system. LC–MS/MS used an Agilent Zorbax SB-C18 column (2.1 × 50 mm, 5 µM, USA) operated at 35 °C, at a flow rate 0.5 mL/min with a gradient consisting of 0.01 % formic acid in MilliQ water (Millipore, USA) and 0.01 % formic acid in 80 % acetonitrile-20 % MilliQ water. The MS1 and MS2 were operated at unit resolution in the multiple reaction monitoring (MRM) mode, monitoring the transition of the protonated molecular ions at m/z 368 to the product ions at m/z 245 for derivative **3** and monitoring the transition of the protonated molecular ions at m/z 372 to the product ions at m/z 245 for derivative **1** (internal standard). No interference of derivative **1** fragment (372 > 245) to derivative **3** fragment (368 > 245) was observed in MRM channel. The lower limit of quantification for derivative **3** in plasma was 0.003 μM.

Mean mouse plasma-time data for derivative **3** was analysed with non-compartmental method using Phoenix WinNonlin 6.2 (Pharsight Corporation, Mountain View, CA) as shown in Table 1.

**Table 1:** The results of the pharmacokinetic study

Parameter	Value
K_e_,1/h	0.017
t_1/2_, h	4.11
C_max_, µmol/L	0.087
AUC_0–6 h_, µmol/L × h	0.13
AUC_0–∞_, µmol/L × h	0.19

*K*_*e*_ elimination rate constant, *t*_*1/2*_ elimination half-life, *C*_*max*_ maximum concentration in plasma, *AUC* area under the plasma concentration–time curve up to 6 h and extrapolated to infinity

## Results

### Thieno[2,3-*b*]pyridine derivatives inhibit cell proliferation

**Fig. 2:**
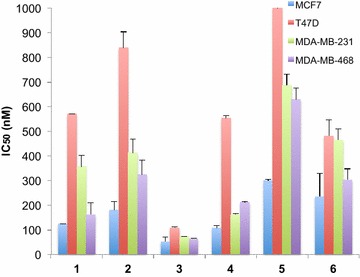
Thieno[2,3-*b*]pyridines derivatives inhibit cell proliferation. Relative thymidine incorporation of a panel of breast cancer cell lines was measured after 3 days treatment of derivatives **1–6**. IC_50_ values (50 % inhibition of growth) are shown in nanomolar (nM). IC_50_ value was not reached for the concentration tested for derivative **5** in T47D cell line

The effect of derivatives **1–6** on the proliferation of four breast cancer cell lines (MCF7, T47D, MDA-MB-231 and MDA-MB-468) was measured using a thymidine uptake assay [19], as shown in Fig. 2. The IC_50_ values were comparable (Spearman Rank Order Correlation with r = 0.708, p < 0.0001) with those previously reported results from the NCI60 human tumour cell line panel on the same cell lines using the sulforhodamine B assay for cell density determination [10–12, 13]. Derivative **3** (NSC768313), with a methoxy substitution at the *meta* position, was the most active; IC_50_ < 100 nM for all the cell lines tested except for T47D and the triple-negative MDA-MB-231, and was chosen for the further studies.

A COMPARE [20] analysis (based on the NCI60 data) of the growth inhibition patterns of different tumour cell lines by the thieno[2,3-*b*]pyridine derivative **3** showed Pearson correlation coefficient (PCC) of 0.512 with a mitotic kinesin Eg5 inhibitor S-trityl-l-cysteine [21], the G2-phase arresting agent cytembena [22] (PCC = 0.456), the microtubule poison vinblastine [23] (PCC = 0.456), and the microtubule poison paclitaxel [24] (PCC = 0.3).

### Thieno[2,3-*b*]pyridine derivative 3 induces G2/M cell cycle arrest but not DNA damage

**Fig. 3:**
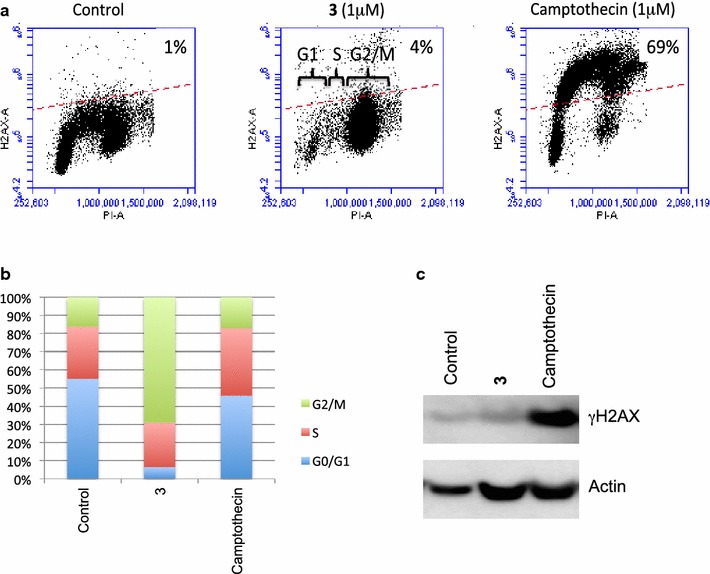
Thieno[2,3-*b*]pyridine induces G2/M cell cycle arrest but not DNA damage. MDA-MB-231 breast cancer cells treated by derivative **3** (1 μM) or camptothecin (1 μM) for 24 h. **a** The effect of DNA damage was shown as relative measure of γH2AX abundance in the MDA-MB-231 breast cancer cells. Cellular immunofluorescence (anti-**γ**-H2AX antibody; *y-axis*) is plotted against DNA content (propidium iodide staining; *x-axis*). The top half (marked by *dashed line*) represents high γ-H2AX phosphorylation and the proportion of the total is indicated. **b** The cell cycle proportions of MDA-MB-231 treated by derivative **3** (1 μM) or camptothecin (1 μM) for 24 h. Population of G2/M phase was significantly increased by derivative **3**. **c** Immunoblot for the phosphorylated **γ**-H2AX in the MDA-MB-231 breast cancer cell line. Actin was used as a loading control

The effect of the derivative **3** on the cell cycle distribution of MBA-MB-231 tumour cells was investigated by flow cytometry. DNA damage was measured by the increase of phosphorylated protein γH2AX, which is a well-established biomarker for DNA double strand breakage [25]. The effects were compared with those of camptothecin as a typical DNA damaging agent. Derivative **3** did not initiate DNA damage comparable to camptothecin (increased in the phosphorylated protein γH2AX from basal level of 1 % to camptothecin treated 69 %) but did induce G2/M phase cell cycle arrest (Fig. 3a, b, c).

### Thieno[2,3-*b*]pyridine derivative 3 induces a multinucleated phenotype but not mitotic arrest

**Fig. 4:**
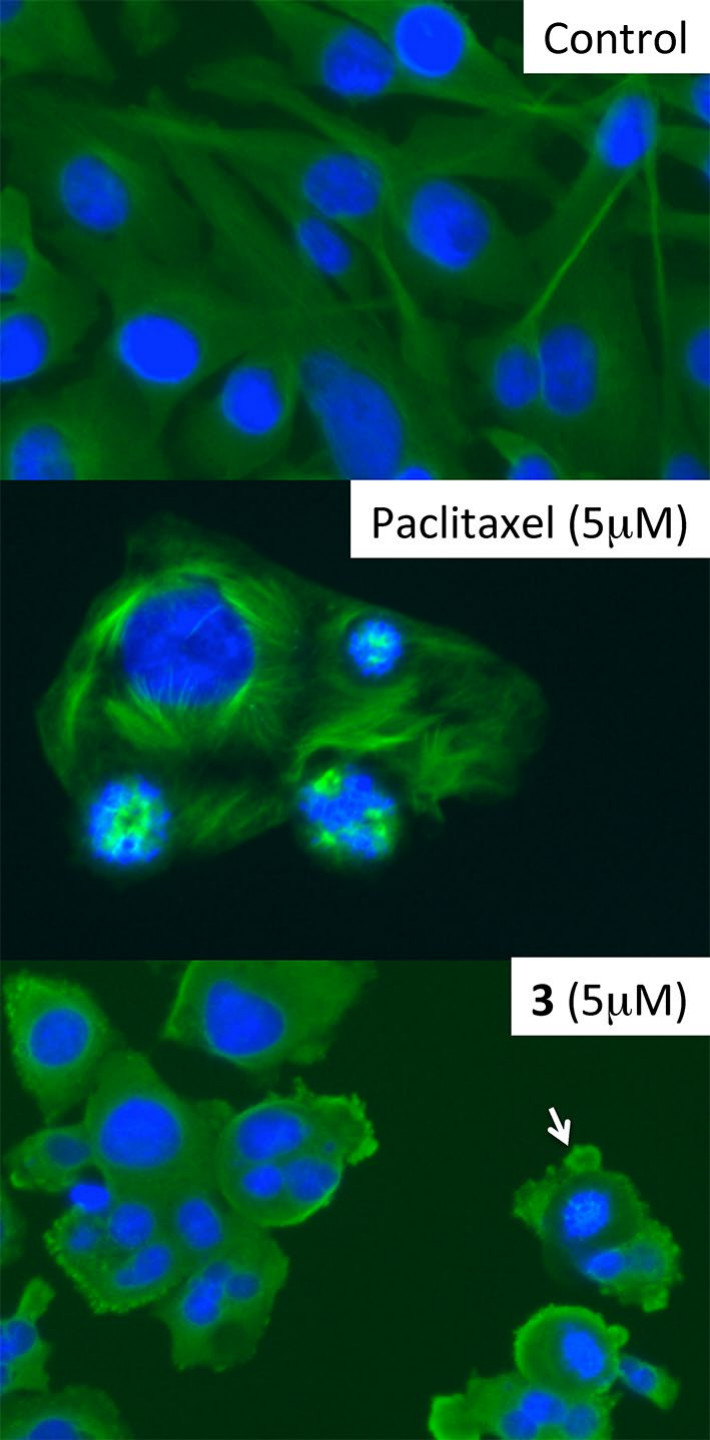
Thieno[2,3-*b*] induce multinucleated phenotype but not microtubules bundle formation. The effect of paclitaxel and derivative **3** on MDA-MB-231 breast cancer cells with microtubule filaments stained with FITC-labelled anti-tubulin antibody (*green fluorescence*). DNA in the nucleus was stained with Hoechst staining (*blue fluorescence*). Cells were treated with or without drugs for 16 h. Multi-nucleated cells were observed by derivative **3** treatment. The *arrow* indicates membrane blebbing

Earlier flow cytometry showed that the thieno[2,3-*b*]pyridine derivative **1** caused G2/M arrest [13], which is also observed with tubulin poisons [26]. We next examined the effects of derivative **3** on microtubule formation in MDA-MB-231. Organised cytoplasmic microtubules complexes radiate from the centromere of the cells is shown in the control MDA-MB-231 cells. Derivative **3** treated cells exhibited abnormal nuclear phenotype and possessed multinuclear cells (Fig. 4). As expected, paclitaxel induced the shortened and bundling of microtubules. No microtubule bundles were observed, suggesting derivative **3** had a different mechanism from that of paclitaxel. Derivative **3** initiated the cells to round up as previously seen [13] but they also induced the formation of membrane protrusions on the cell surface (membrane blebbing).

### Thieno[2,3-*b*]pyridine derivative 3 has no effect on calcium flux

**Fig. 5:**
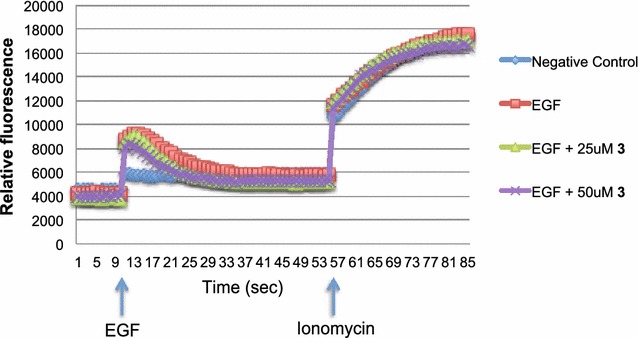
Thieno[2,3-*b*]pyridine has no effect on calcium flux. Calcium flux measured in A431 cells with and without pre-treatment of derivative **3**. EGF (400 ng/ml) was added at the time marked by the first *vertical arrow*, followed by ionomycin (1 µg/ml) as indicated, as a positive control

It is established that PLC-γ isoforms are involved in regulating calcium ion flux while PLC-δ1 isoform is a calcium response element operating downstream of other PLC’s [8, 27]. To examine whether derivative **3** could reduce the Ca^2+^ mobilization in the cell cytosol, we examined the response of squamous carcinoma A431 cells by adding epidermal growth factor (EGF) following pre-treatment with derivative **3** at 25 and 50 μM (Fig. 5). The A431 cell line was chosen because it has been previously used to monitor calcium concentration for the thieno[2,3-*b*]pyridines [2]. Derivative **3** had no effect on the increase in intracellular calcium levels induced by EGF.

### Interactions between thieno[2,3-*b*]pyridine derivative 3 and cytotoxic drugs

**Fig. 6:**
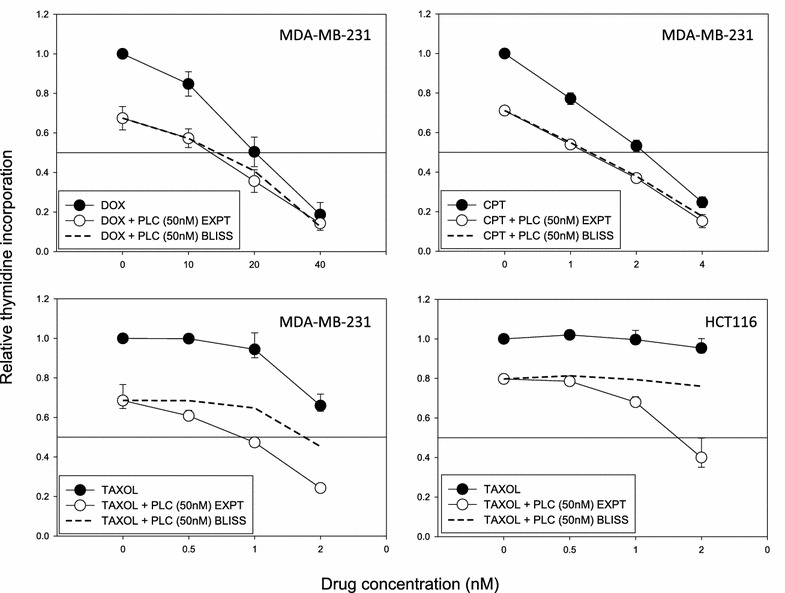
Synergy using thieno[2,3-*b*]pyridine with cytotoxic drugs. The growth inhibitory effects of derivative **3** (50 nM) in combination with cytotoxic drugs on breast cancer cell line MDA-MB-231 and the colon breast cancer cell line HCT116. An average of two independent experiments is shown. *CPT* camptothecin, *DOX* doxorubicin, *TAXOL* paclitaxel; and PLC, derivative **3**. The *dashed line* represents the theoretical expectation when the combined effects of chemicals are additive (*Bliss curve*)

The ability of the thieno[2,3-*b*]pyridine derivative **3** to modulate the effects of the cytotoxic drugs doxorubicin, camptothecin or paclitaxel was explored in MDA-MB-231 cells. The Bliss independence model [28] was used to assess drug interactions. The topoisomerase I poison camptothecin and the topoisomerase II poison doxorubicin, which target topoisomerase I and II, respectively, showed only additive effects with derivative **3** but for paclitaxel a synergistic effect is seen. Synergism with paclitaxel and derivative **3** was also observed in colon cancer cell line HCT116 (Fig. 6).

### Thieno[2,3-*b*]pyridine derivative 3 induces acceptable growth inhibitory plasma concentrations in mouse plasma

**Fig. 7:**
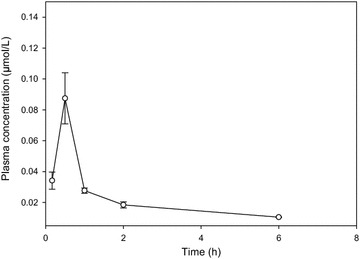
Plasma concentrations in CD1 male mice as a function of time after administration of derivative **3.** Mice, (n = 3); derivative 3, (10 mg/Kg)

Derivative **3** was chosen for a small pharmacokinetic study. It showed poor aqueous solubility and was therefore formulated in 20 % hydroxypropyl β cyclodextrin (HP-β CD), resulting in a fine yellow suspension. This was administered at a dose of 10 mg/Kg by intraperitoneal injection and was well tolerated with no acute reaction observed. The plasma concentration is shown as a function of time in Fig. 7 and pharmacokinetic data are shown in Table 1. The maximal plasma concentration achieved in mice (30 min post injection) was 0.087 μmol/L and the half-life was over 4 h.

## Discussion

Our results provide new information on mechanism of the thieno[2,3-*b*]pyridines and their potential for development in anticancer therapy. We have identified NSC768313 (derivative **3**) as a potent derivative in this series and shown that it inhibits the proliferation of two triple negative human breast cancer cell lines, as well as one of two oestrogen receptor positive cell lines, at a concentration of <100 nM. The derivative causes morphological changes including membrane blebbing, as well as inducing G2-phase arrest. The results appear to rule out DNA damage, mitotic arrest and increased calcium ion flux as mechanisms of growth inhibition.

Three results suggest that the thieno[2,3-*b*]pyridines act on events leading to mitosis. Firstly, results from flow cytometry and fluorescence microscopy indicate arrest in the G2-phase rather than metaphase. Secondly, COMPARE analysis suggests that the growth inhibitory effects are related in some way to those of the mitotic poisons vinblastine and paclitaxel. However, they are also related to those of S-trityl-l-cysteine and cytembena, which induce G2-phase arrest but not mitotic arrest. Thirdly, derivative **3** shows strong synergy with paclitaxel but not with doxorubicin and camptothecin in their growth arrest of MDA-MB-231 cells.

The cellular effects of the thieno[2,3-*b*]pyridine derivative **3** are comparable to those observed in MDA-MB-231 breast cancer cells following PLC–δ1 and –δ3 gene knockdown [8], which induces severe growth inhibition and G2-phase cell cycle arrest. The effects are also similar to those of the thieno[2,3-*b*]pyridine derivative **1** [8, 13]. Phosphatidylinositol 4,5-bisphosphate (PIP2) coordinates actin polymerisation and the formation of the filopodia membrane for cell adhesion [29]. Since PLC–δ1 binds directly to PIP2, the change of cell morphology (cell rounding and membrane blebbing) supports the hypothesis that thieno[2,3-*b*]pyridines act as PLC–δ1 inhibitors. It is of interest that PLC1, the yeast homologue of PLC-δ1, localizes at the centromeric loci at the G2/M checkpoint and is essential for cell growth [8]. There is evidence that PLC–γ interacts directly with β-tubulin through pleckstrin homology domains [30], which are also present in PLC–δ1 [31]. We were unfortunately not able to test the effects of this series of thieno[2,3-*b*]pyridines on PLC–δ1 in a cell-free assay. The compounds were tested for PLC-β/γ inhibition; no effect was found for PLC-β and the order of potency for the inhibition of PLCγ was different from that for inhibition of cell proliferation (data not shown). We suggest that active thieno[2,3-*b*]pyridine derivatives interact with PLC isozymes, which then inhibit the function of the cytoskeleton. Changes to tubulin lead to aberrant mitoses and to subsequent multinucleate cells and synergy with paclitaxel. Changes to actin may also contribute to the observed morphological changes.

Preliminary experiments show that derivative **3** has acceptable pharmacological properties. Although a preliminary mouse xenograft study showed reduction of tumour size and mass upon intraperitoneal dosing with thieno[2,3-*b*]pyridine derivative **4** (Fig. 1), the results did not reach statistical significance [32]. Future work will focus on finding new analogues in this series with increased aqueous solubility as well as expanding structure–activity relationships (SAR) for the series. More effective analogues, in conjunction with molecular modelling studies, could then be used to evaluate in vivo antitumour activity as well as identifying the biochemical target of action.
